# Validity of mini-fluid challenge for predicting fluid responsiveness following liver transplantation

**DOI:** 10.1186/s12871-019-0728-4

**Published:** 2019-04-13

**Authors:** Ahmed Mukhtar, Maha Awad, Mohamed Elayashy, Amr Hussein, Gihan Obayah, Akram El Adawy, Mai Ahmed, Hisham Abul Dahab, Ahmed Hasanin, Amr Elfouly, Mostafa Abdo, Amr Abdelaal, Jean Louis Teboul

**Affiliations:** 1 Department of Anesthesia, Surgical Intensive Care and Pain Management, Faculty of Medicine, Cairo University, Cairo, Egypt; 2 Department of Critical Care, Faculty of Medicine, Cairo University, Cairo, Egypt; 3 Department of Internal Medicine, Faculty of Medicine, Helwan University, Cairo, Egypt; 4 Department of Surgery, Ainshams University, Cairo, Egypt; 5 Medical ICU, Bicêtre Hospital, Paris-South University, Paris, France; 6 Cairo, Egypt

**Keywords:** Minifluid challenge, Liver transplantation, Fluid responsiveness

## Abstract

**Background:**

Mini-fluid challenge is a well tested and effective tool to predict fluid responsiveness under various clinical conditions. However, mini-fluid challenge has never been tested in patients with end-stage liver disease. This study investigated whether infusion of 150 ml albumin 5% can predict fluid responsiveness in cirrhotic patients following liver transplant.

**Methods:**

Fifty patients receiving living donor liver transplant were included in the analysis. Mini-fluid challenge composed of 150 ml of albumin 5% administered over 1 min in three consecutive 50-ml fluid boluses. An additional 350 ml was then infused at a constant rate over 15 min (for a total of 500 ml). Stroke volume (SV) was measured as the product of the subaortic velocity time integral (VTI) and left ventricular outflow tract (LVOT) area. Fluid responsiveness was defined as an increase in SV by ≥15% after the infusion.

**Results:**

Fifty patients were enrolled in the study. Fourteen patients were classified with Child A, 15 patients with Child B, and 21 patients with Child C cirrhosis. Thirty four patients were fluid responders and 16 patients were fluid non-responders. After 150 ml of albumin 5%, the SV increased significantly in our cohort. The area under receiver operating curve (AUROC) was 0.7 (95% confidence interval [CI] 0.5–0.8, *P* = 0.005). In subgroup analysis, the SV increased significantly after mini fluid challenge in the Child A group (*P* = 0.017) but not Child B or C groups (*P* = 0.3 and 0.29, respectively). The AUROC for mini-fluid challenge in the Child A group was 0.86 (95% confidence interval [CI] 0.6–0.9, *P* = 0.0004), while mini-fluid challenge failed to discriminate between responders and non-responders in Child B and C groups.

**Conclusion:**

A mini-fluid challenge of 150 ml albumin 5% can predict fluid responsiveness in liver transplant patients with fair sensitivity and specifiicty. Subgroup analyis revealed that minifluid challenge can predict fluid responsiveness in patients with Child A cirrhosis but not patients with Child B or C cirrhosis.

**Trial registration:**

NCT03396159. (Prospective registered). Initial registration date was 10/01/2018.

## Background

Hemodynamic derangements are frequently observed during and after liver transplantation, mainly due to major blood loss, decreased venous return, and (or) reperfusion injury-induced vasodilatation. Fluid therapy is the cornerstone of hemodynamic management for unstable patients. The main goals of fluid therapy are to increase cardiac output (CO) and improve tissue perfusion.

The concept of fluid responsiveness has been suggested as a guide for fluid administration in critically ill patients to avoid over- and under-fluid resuscitation. Fluid responsiveness is defined as the ability of the left ventricle to increase stroke volume (SV) after fluid administration [1, 2]. Several methods have been suggested to detect fluid responsiveness. Of these methods, some that depend on heart−lung interactions have been shown to be effective during intraoperative management of liver transplantation patients. However, during the postoperative period, these methods are not feasible in patients with spontaneous breathing activity. In these cases, mini-fluid challenge has emerged as an alternative for preload challenge [3].

Several studies have investigated the value of mini-fluid challenge as a predictor of subsequent fluid infusion under different clinical conditions [4, 5]. However, none of these studies included patients with end-stage liver disease ESLD. Patients with ESLD have abnormal blood volume distribution, with a substantial fraction of this volume in the splanchnic circulation [6]. Rapid volume loading in these patients has little impact on CO because a large proportion of infused fluid is shifted to the splanchnic system. As a result, aggressive volume expansion to maintain tissue perfusion in liver transplantation recipients may be associated with volume overload and increased risks of poor graft function and postoperative morbidity [7]. Moreover, not all patients with ESLD exhibit the same response to changes in blood volume. Healthy individuals and patients with Child A cirrhosis differ in their response to blood volume expansion compared to patients with Child B and C cirrhosis [8].

The primary objective of the present study was to investigate the validity of mini-fluid challenge as a predictor of fluid responsiveness in patients with ESLD after liver transplantation. The seconadry objective was to examine the consistency of this validity among different Child classification. Our hypothesis was that mini-fluid challenge would not be a valid tool to predict fluid responsiveness in cirrhotic patients after liver transplant.

## Methods

This prospective observational study was designed to enroll 50 consecutive living donor liver transplant (LDLT) patients. The indications for LDLT in our center include; patients with decompensated liver cirrhosis, liver cirrhosis with MELD score more than 15, and patients with hepatocellular carcinoma within transplant criteria. The study protocol was approved by the Research Ethics Committee of Cairo University Hospital and informed consent was obtained from all patients. The study is registered at clinical trial.gov (NCT03396159) and conducted from January 2018 to May 2018. Patients < 18 years of age, poor echocardiographic window, patient with CVP > 12 or lung congestion or those with acute fulminant liver failure were excluded from the study.

Before ICU admission, a 20G radial artery catheter was inserted to measure arterial pressure and a triple lumen central venous catheter was inserted into the right jugular vein to measure central venous pressure (CVP). We typically used a fast track protocol, and all patients were extubated at the end of the operation inside the operating room.

Upon admission to the intensive care unit (ICU), patients were maintained in a supine position and pressure transducers were referenced to the level of the tricuspid valve and zeroed to atmospheric pressure after calibration.

### Study protocol

All measurements were performed in the supine position within 1 h after arrival in the ICU. Hemodynamic measurements were performed at baseline. The fluid challenge was administered intravenously via a specific venous line. Mini-fluid challenge consisted of 150 ml albumin 5% delivered over 1 min as three consecutive 50-ml fluid boluses with no time delay between them. The remaining 350 ml was infused at a constant rate using preset electrical infusion pump over 15 min (for a total infusion volume of 500 ml). Three sets of SV measurements were obtained: The first set was obtained before the mini-fluid challenge, the second set 1 min after the mini-fluid challenge, and the third set immediately after the infusion of the remaining 350 ml (total = 500 ml) given over 15 min. Fluid responsiveness was defined as an increase in SV by 15% after the infusion of 500 ml albumin 5%, thus separating the studied population into responders and non-responders. This cut of value was chosen as described previously [5].

Echocardiographic assessment was performed by an accredited physician (level 3) using a GE Vivid E9 echocardiographer. The assessor was blinded to Child-Pugh class of the patients. Stroke volume was measured as the product of the subaortic velocity time integral (VTI) and left ventricular outflow tract (LVOT) diameter. The VTI was obtained by placing the pulse wave Doppler sampling cursor in the middle of the LVOT immediately proximal to the aortic valve in the apical five-chamber view. For each step of the study, VTI was measured in triplicate and averaged for the determination of the VTI value. The LVOT diameter was measured for each patient as the distance between the inflection points at the base of the aortic valve cusps from the left parasternal long-axis view during systole. Finally, SV was calculated as VTI × LVOT area.

Primary endpoint in this study was the sensitivity and specificity of minifluid challenge to assess fluid responsiveness in cirrhotic patients.

### Statistical analysis and sample size calculation

Sample size was calculated to detect an area under the receiver operating curve (AUROC) of 0.75 with null hypothesis AUROC = 0.5. Considering that the ratio of responders admitted to our unit is about half that of non-responders, a minimum of 42 patients including at least 14 responders are needed for a study power of 80% and an alpha error of 0.05.

Data are expressed as mean (with standard deviation) unless specified otherwise. Continuous variables were compared by one-way ANOVA and post hoc Tukey tests, while categorical variables were compared by χ^2^ test. Paired variables within each group (before and after mini-fluid challenge) were compared using the Wilcoxon matched-pairs test. To compare the performance of mini-fluid challenge for assessing fluid responsiveness among subgroups (Child cirrhosis classes), receiver operating characteristic (ROC) curves were constructed and the area under the curve (AUC) calculated. MedCalc version 12.1.4.0 (MedCalc Software bvba, Mariakerke, Belgium) generated values with the highest sensitivity and specificity (Youden index). All statistical analyses were performed using SPSS for Windows version 15.0 (SPSS Inc., Chicago, IL, USA).

## Results

Out of 60 patients screened, 55 met the inclusion criteria and fifty patients were available for final analysis. The reason for exclusion in all cases was a poor echocardiographic window. Fourteen patients had Child A cirrhosis, 15 Child B, and 21 patients Child C cirrhosis. Age, sex, and BMI were comparable among the three groups. However, Model for End-Stage Liver Disease (MELD) score was significantly lower in the Child A group compared to both Child B and C groups (Table 1). All patients had preoperative good cardiac function with estimated ejection fraction between 55 and 65%. Two patients with child C cirrhosis had history of preoperative ICU admission and none of studied population required renal replacement therapy before liver transplantation. All patients were admitted to ICU after successful extubation inside the operating room. The intra-observer variability was 5%. Thirty four patients were fluid responders and 16 patients were fluid non-responders. After 150 ml of albumin 5%, the SV increased significantly in our cohort. The AUROC was 0.7 (95% confidence interval [CI] 0.5–0.8, *P* = 0.005). and the optimum cut-off value for predicting fluid responsiveness was ≥10%. This cut-off value had 56% sensitivity and 87% specificity for distinghishing responders from non-responders. In subgroup analysis, the SV increased significantly in the Child A group (*P* = 0.017) but not Child B or C groups (*P* = 0.3 and 0.29, respectively) after mini fluid challenge. All other hemodynamic variables remained unchanged during and following mini-fluid challenge and after completion of 500 cc albumin 5% (Tables 2 and 3). After fluid challenge, the SV increased by more than 15% in 10 patients of the Child A group (71.4%), 11 patients in the Child B group (73.3%), and 13 patients in the Child C group (62%). The AUC for mini-fluid challenge in the Child A group was 0.86 (95% CI 0.6–0.9, *P* = 0.0004) and the optimum cut-off value for predicting fluid responsiveness was ≥10%. This cut-off value had 70% sensitivity and 100% specificity for distinghishing responders from non-responders. In contrast to the Child A group, mini-fluid challenge failed to discriminate between responders and non-responders in the Child B and C groups (Table 4). The change in CVP after 150 cc loading did not discriminate between responders and non-responders in any of the three groups.

**Table 1:** Patient characteristics

	All patients (*n* = 50)	Child A (*n* = 14)	Child B (*n* = 15)	Child C (*n* = 21)	*P*-value
Age (years)	52 (45–57)	51 (45–57)	52 (42–55)	56 (48–60)	0.23
Sex M/F	46/4	14/0	13/2	19/2	0.39
BMI	26 (24–29)	27 (23–29)	26 (23–26)	26 (24–29)	0.35
MELD	13 (11–17)	10 (9–11)*	16 (12–17)	17 (13–19)	< 0.001
Graft weight (g)	835 (660–950)	865 (615–1022)	690 (620–800)†	870 (740–990)	0.012
Intraoperative use of vasopressor	20 (40%)	0	2 (13%)	18 (86%)*	< 0.001
Intraoperative use of colloid (ml)	2000 (1000)	1500 (500)	1700 (650)	2000 (900)	0.14
ICU stay (days)	7 (7–10)	7 (7–10)	7 (7–14)	7 (7–10)	0.9
Hospital stay (days)	21 (21–21)	21 (21–22)	21 (21–30)	21 (21–21)	0.78

Data are presented as median (IQR) or ratio

*BMI* body mass index, *MELD* Model for end-stage liver disease, *ICU* intensive care unit

^a^denotes significant compared to the other two groups. ^b^denotes significant compared to CHILD C. *p* < 0.05

**Table 2:** Hemodynamic variables at baseline and after mini-fluid challenge

	Child A (*n* = 14)			Child B (*n* = 15)			Child C (*n* = 21)		
	Baseline	After mini-fluid challenge	*P* value	Baseline	After mini-fluid challenge	*P* value	Baseline	After mini-fluid challenge	*P* value
HR	106 (94–115)	109 (89–115)	0.3	103 (84–114)	100 (86–113)	0.08	102 (86–113)	100 (93–113)	0.48
CVP	6 (3–8.5)	6 (2.5–9.5)	0.4	7 (5–9)	7 (6–11)	0.06	5 (4–8)	5 (4–8)	0.1
MAP	107 (94–112)	101 (94–117)	0.9	86 (80–93)	98 (82–100)	0.08	95 (83–104)	98 (97–103)	0.9
SV	87 (60–149)	101 (85–147)	0.017	89 (70–132)	102 (72–148)	0.3	90 (63–115)	91 (80–118)	0.29

**Table 3:** Hemodynamic variables at baseline and after completion of 500 cc fluid challenge

	Child A (*n* = 14)			Child B (*n* = 15)			Child C (*n* = 21)		
	Baseline	After fluid challenge	*P* value	Baseline	After fluid challenge	*P* value	Baseline	After fluid challenge	*P* value
HR	106 (94–115)	104 (89–111)	0.3	103 (84–114)	103 (86–110)	0.09	102 (86–113)	98 (88–110)	0.45
CVP	6 (3–8.5)	5 (4–9.5)	0.8	7 (5–9)	9 (5–12)	0.06	5 (4–8)	6 (5–8)	0.15
MAP	107 (94–112)	101 (95–119)	0.9	86 (80–93)	93 (83–103)	0.07	95 (83–104)	96 (86–102)	0.5
SV	96 (58–152)	111 (94–153)	0.002	89 (68–136)	114 (92–165)	0.004	90 (63–115)	110 (81–132)	0.001

**Table 4:** Comparison of the ability of mini-fluid challenge to assess fluid responsiveness among the three groups

	AUC of ROC curve	95% CI	Cut-off value	Sensitivity (%)	Specificity (%)	*P*-value
Child A	0.86*	0.6–0.9	10%	70	100	0.0004
Child B	0.7	0.4–0.9	16%	100	50	0.26
Child C	0.6	0.4–0.8	7%	58	87	0.2

*P* value denotes difference of AUC vs AUC of 0.5

*AUC* area under the curce, *ROC* receiver operating characteristic

*denotes statistical significance (*P* < 0.05)

## Discussion

The main finding of the present study is that a mini-fluid challenge (150 ml of albumin 5%) is sufficient to assess fluid responsiveness in patients with Child A cirrhosis but not Child B or C cirrhosis.

We found that a SV change > 10% during mini-fluid challenge following liver transplant asess fluid responsiveness in Child A cirrhosis patients with 70% sensitivity and 100% specificity. To the best of our knowledge, this is the first study to investigate mini-fluid challenge testing in patients with ESLD. Patients with ESLD not only have an abnormally high blood volume, but also an abnormal blood volume distribution. However, not all patients with ESLD exhibit the same response to changes in blood volume. Healthy individuals and patients with Child A cirrhosis differ in their response to blood volume expansion compared to patients with Child B and C cirrhosis. Volume expansion resulted in significantly increased central blood volume in patients with Child A cirrhosis compared to patients with Child B or C cirrhosis, [8] which may explain the difference in patient response to mini-fluid challenge observed in the present study. The plausible mechanism of this finding was that patients with Child A have less severe alteration in vasomotor and venous capicitance compared to Child B and C [9]. Thus, mini fluid bolus (150 mL) could increase the mean systemic filling pressure in patients with Child A but not in patients with Child B or C.

In the early stages of our liver transplant program, we typically extubated the patient 6–8 h after admission to the ICU [10]. However, we changed our protocol 8 years ago and more than 90% of patients are now extubated successfully inside the operating room. This makes the assessment of fluid responsiveness difficult because all dynamic indices, including pulse pressure variation and stroke volume variation, are unreliable during spontaneous breathing. Mini-fluid challenge and passive leg raising remain the only available tests for spontaneously breathing patients.

There is currently great interest in using fluid responsiveness as determined by mini-fluid challenge to guide transfusion, particularly to avoid volume overloading. While several studies have tested the concept of mini-fluid challenge, these investigations used different volumes and fluid types [7, 11, 12]. In the first such study, Muller et al. investigated the validity of mini-fluid challenge among 39 critically ill ventilated patients with acute circulatory failure and found that an increase in aortic blood flow ≥10% after rapid infusion of 100 ml hydroxyethyl starch predicted fluid responsiveness.^7^ More recently, Aya et al. found that 4 ml/kg was the minimum volume required to evoke a significant hemodynamic effect [10]. Smorenberg et al. reported that 150 ml of hydroxyethyl starch reliably predicted fluid responsiveness after cardiac surgery [13]. In our study, we used the same volume (150 ml) of albumin 5% and found this amount to be effective only in patients with Child A cirrhosis. Thus, a higher-volume mini-fluid challenge may be required for patients with advanced cirrhosis.

The concept of using predefined criteria for increase of CO after fluid challenge to differentiate between responder and non-responder depends on the starling mechanism. However, it is worth mentioning that CO changes during fluid bolus is affected not only by preload but also by afterload. Rapid fluid infusion decrease systemic vascular resistance by hemodilution. One study found that infusion of 1 l of colloid caused 20% increase in blood volume, 20% decrease in hemoglobin, and 40% increase in CO [14]. The increase in CO in their study is probably due to not only an increase in preload but also a decrease in afterload.

In our study, we defined fluid responsiveness as an increase in SV by 15% after infusion of 500 ml albumin 5%. Despite that the concept of fluid responsiveness has been emerged 20 years ago, there are still considerable variabilities in the definition, implementation, and assessment of fluid challenge. Recent systematic review found that 60% of the studies defined fluid responsiveness as an increase of ≥15% of CI or CO immediately after fluid challenge [15].

Albumin 5% infusion was chosen to test fluid responsiveness instead of hydroxyethyl starch because we have previously shown that this solution is as effective for volume expansion [16]. Moreover, the safety of hydroxyethyl starch has been questioned and we have abandoned its use in the postoperative period [17].

Our study had several limitations. First, all our patients had excellent cardiac function during the early. Postoperative period, so we cannot extrapolate our findings to patients with poor cardiac function. Second, assessment of minifluid using echocardiography is challenging because the minifluid challenge induces small changes in cardiac output which may miss some true responders, however, we used single operator to limit the effect of interobserver variability.

## Conclusion

A mini-fluid challenge of 150 ml albumin 5% can predict fluid responsiveness in liver transplant patients with fair sensitivity and specifiicty. Subgroup analyis revealed that minifluid challenge can predict fluid responsiveness in patients with Child A cirrhosis but not in patients with Child B or C cirrhosis.

## Abbreviations

AUROC: Area under receiver operating curve
CO: Cardiac output
CVP: Central venous pressure
ESLD: End stage liver disease
ICU: Intensive care unit
LVOT: Left ventricular outflow tract
MELD: Model for end stage liver disease
SV: Stroke volume
VTI: Velocity time integral
